# Functional DNA Repair Profiling in Translational Medicine: Benchmarking Comet, γH2AX, and NGS Assays Against Clinical Constraints

**DOI:** 10.3390/cimb48020149

**Published:** 2026-01-29

**Authors:** Anna Macieja, Marta Poplawska, Karolina Przybylowska-Sygut, Joanna Makowska, Tomasz Poplawski

**Affiliations:** 1 Department of Pharmaceutical Microbiology and Biochemistry, Medical University of Lodz, 92-215 Lodz, Poland; anna.macieja@umed.lodz.pl (A.M.); karolina.przybylowska@umed.lodz.pl (K.P.-S.); 2 Biobank, Department of Immunology and Allergy, Medical University of Lodz, 92-213 Lodz, Poland; marta.poplawska@umed.lodz.pl; 3 Department of Rheumatology, Medical University of Lodz, 92-115 Lodz, Poland; joanna.makowska@umed.lodz.pl

**Keywords:** DNA repair, comet assay, γH2AX, NGS, PBMC, biobanking, autoimmune disease

## Abstract

Quantifying DNA repair capacity (DRC) is pivotal for stratifying patients in oncology and autoimmune disorders, yet methodological heterogeneity compromises data reproducibility. While basic research relies on genetically encoded reporters, translational settings demand robust assays compatible with biobanked material, particularly Peripheral Blood Mononuclear Cells (PBMCs). This review benchmarks functional DNA repair assays—ranging from alkaline/neutral comet variants and high-content foci imaging (γH2AX/53BP1) to emerging Next-generation sequencing (NGS)-based break mapping—against the rigors of clinical application. We critically evaluate sensitivity, specificity, and throughput, identifying artifacts introduced by cryopreservation, steroid therapy, and oxidative stress. Furthermore, we propose a “Minimum Reporting Standard” checklist to harmonize DRC quantification. By distinguishing established validation tools from experimental artifacts, this framework aligns assay selection with specific biological endpoints and clinical feasibility.

## 1. Introduction: The Translational Gap in DNA Repair Profiling

Genomic instability drives both oncogenesis and the therapeutic vulnerability exploited by DNA-damaging agents. Patient stratification for platinum-based chemotherapy or PARP inhibitors currently relies on static genomic biomarkers—Breast Cancer Gene 1/2 (BRCA1/2) mutations being the canonical example [1,2,3]. Yet genotype does not equal phenotype. A truncating mutation may retain partial function [4]; a wild-type sequence may be silenced epigenetically [5] or rendered irrelevant by proteomic bottlenecks elsewhere in the pathway [6]. Predicting therapeutic response therefore demands direct, functional measurement of DNA repair capacity (DRC), the cell’s ability to resolve genotoxic lesions, assessed through challenge-recovery kinetics, not inference from sequence alone.

The molecular architecture of the DNA damage response (DDR)—Base Excision Repair (BER), Nucleotide Excision Repair (NER), Double-Strand Break (DSB) Repair—is textbook material [7]. Translating this knowledge into clinical assays is not. The bottleneck is rarely conceptual; it is logistical and biological. Techniques optimized for immortalized cell lines, such as plasmid-based host cell reactivation, routinely fail in primary human cells due to poor transfection efficiency and the quiescent metabolism of freshly isolated mononuclear cells [8,9,10].

Why, then, focus on Peripheral Blood Mononuclear Cells (PBMCs)? The answer is pragmatic, not ideal. PBMCs represent the only nucleated, viable cell population routinely accessible from patients without invasive biopsy. In oncology, PBMCs serve as a surrogate tissue reflecting systemic genotoxic exposure and germline repair capacity—they do not recapitulate tumor-intrinsic DDR rewiring. Still, they capture a constitutional repair capacity that determines normal tissue toxicity and may predict tolerance to dose-intensive regimens [11]. In autoimmune and inflammatory diseases, PBMCs are not surrogates; they are the pathophysiologically relevant effector population, and their repair status directly informs the disease mechanism [12,13,14]. This dual role—surrogate in one context, target tissue in another—makes PBMCs a uniquely tractable, if imperfect, substrate for functional DRC profiling.

The rationale for bridging oncology and autoimmunity is mechanistic, not merely opportunistic. Both contexts converge on a shared biology of genomic stress [14]. In malignancy, exogenous DNA-damaging agents—platinum compounds, alkylators, Poly(ADP-Ribose) Polymerase (PARP) inhibitors—are deployed to exploit tumor-intrinsic repair defects. In systemic autoimmune disease, the damage is endogenous: chronic inflammation generates reactive oxygen species (ROS) at levels comparable to low-dose ionizing radiation, imposing a continuous repair burden on circulating lymphocytes [15]. The same drugs cross both fields: cyclophosphamide and methotrexate anchor treatment protocols in aggressive lupus and rheumatoid arthritis (RA) just as they do in lymphoma. Corticosteroids, ubiquitous in autoimmune management, independently suppress DNA repair gene expression—a confounder rarely acknowledged in either literature. PBMCs thus occupy a unique position: in oncology, they serve as a surrogate for systemic genotoxic exposure; in autoimmunity, they are both the effector population and the victim of sustained oxidative assault [16,17]. Measuring DRC in this single, accessible compartment provides a unified readout across otherwise disparate clinical domains.

This review does not recatalogue the molecular hierarchy of DDR proteins. Instead, it benchmarks the operational trade-offs among three assay classes applicable to biobanked material: the sensitivity of comet assay variants, the pathway specificity of γH2AX/53BP1 foci imaging, and the nucleotide-resolution mapping offered by emerging Next-generation sequencing (NGS)-based methods (END-seq, Breaks Labeling In Situ and Sequencing (BLISS)). Purely mechanistic models—such as yeast genetics and bacterial two-hybrid screens—fall outside this scope; the focus is on assays deployable in human translational studies. Cytogenetic endpoints—micronucleus formation (CBMN) and metaphase chromosome aberration analysis—detect mutagenic outcomes of unrepaired or misrepaired lesions but do not resolve repair kinetics. These assays fall outside this review’s focus on functional, challenge-recovery profiling and are therefore not discussed further. Beyond surveying available methods, this review proposes a Minimum Information for DNA Repair Profiling (MIDRP) checklist to standardize pre-analytical documentation and enforce the methodological discipline required for clinical translation.

**Figure 1:**
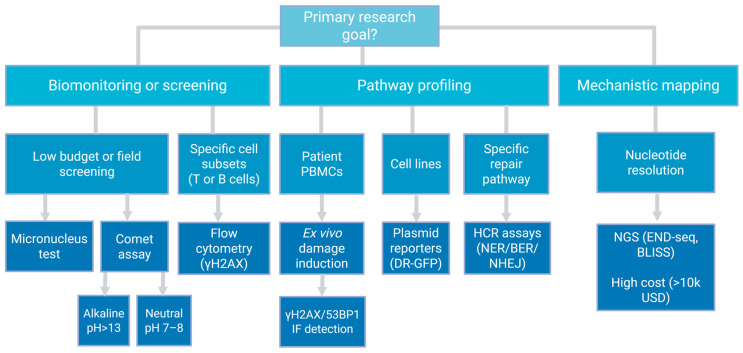
Methodological decision tree for translational DNA repair profiling. Selection of the appropriate assay depends on the primary research goal, sample availability, and budget constraints. Biomonitoring requires high-throughput, low-cost assays (Comet, Flow Cytometry) suitable for large cohorts but offering lower resolution. Pathway Profiling necessitates challenge-recovery designs or specific reporters to assess functional kinetics. Mechanistic Mapping utilizes nucleotide-resolution NGS methods (END-seq, BLISS), which, despite their precision, remain restricted to research settings due to high cost and input requirements (>10^5^–10^6^ cells). Created in BioRender. Macieja, A. (2026) https://BioRender.com/atb11nx.

A recurring theme is pre-analytical vulnerability. Corticosteroid therapy, circulating inflammatory mediators, and ex vivo ischemia do not merely add noise—they systematically bias DRC estimates in directions that mimic or mask actual biological defects [18,19,20]. These confounders are particularly treacherous in autoimmune cohorts, where immunosuppressive treatment is the rule rather than the exception. This review is therefore deliberately prescriptive rather than merely descriptive. Beyond surveying available methods, it identifies the methodological discipline required for clinical translation and proposes a reporting framework to enforce it. The final sections propose a Minimum Reporting Standard and a decision framework (Figure 1) intended to help investigators distinguish genuine repair deficiency from methodological artifact. Without such discipline, functional biomarkers will remain a research curiosity rather than a clinical tool. Throughout this review, we use DRC to denote the functional ability of cells to resolve genotoxic lesions, measured through challenge-recovery kinetics. This dynamic, kinetic assessment differs fundamentally from measuring static baseline damage (accumulated lesions at a single timepoint) or DDR signaling (γH2AX phosphorylation, which reports chromatin remodeling rather than physical repair completion). DRC quantifies the repair process itself—the endpoint that determines clinical outcomes.

## 2. The Gold Standard Revisited: Comet Assay Variants and Applications

Single Cell Gel Electrophoresis (SCGE)—the comet assay—remains the workhorse of genotoxicity testing three decades after its introduction [21]. High-throughput sequencing has not displaced it. The reason is biological, not nostalgic: the comet assay resolves damage at single-cell resolution, exposing the heterogeneity that bulk methods erase. A Western blot for γH2AX returns a population average; SCGE reveals the long tail of hyper-damaged cells—the subpopulation that determines clonogenic survival after chemotherapy or the outlier lymphocytes driving autoimmune flares. This granularity comes at a cost. The assay’s apparent simplicity—embed cells, lyse, electrophorese, score—conceals technical variables that routinely destroy inter-laboratory reproducibility [22]. A note on terminology: this review distinguishes three related but distinct constructs. DRC is the umbrella term for a cell’s ability to resolve genotoxic lesions—it is what we ultimately wish to measure. Repair kinetics refers to the temporal profile of damage resolution, typically expressed as a curve or its summary metrics (area under the curve (AUC), t½). Repair efficiency denotes the fraction of induced damage resolved within a defined time window, expressed as a percentage. The terms are related but not interchangeable: kinetics describes the trajectory, efficiency quantifies the endpoint, and capacity encompasses both.

### 2.1. Alkaline vs. Neutral: What You Detect Depends on What You Dissolve

Buffer pH determines biological scope. The alkaline protocol (pH > 13) denatures the double helix, converting alkali-labile sites into frank breaks and reporting a composite of SSBs, DSBs, and abasic lesions [23,24,25]. Sensitivity is high, specificity is not. A dramatic tail in an alkaline comet may reflect lethal chromosomal fragmentation—or transient BER intermediates, or simple chromatin relaxation in cells undergoing apoptosis [26,27]. Over-interpretation is endemic in the literature.

The neutral variant preserves the duplex structure and is widely believed to detect DSBs selectively. This assumption warrants caution. As Collins and others have noted, SSBs in proximity on opposite strands, relaxed supercoiled loops, and variations in lysis stringency can all produce comet tails under neutral conditions without frank DSB-age [28,29]. The neutral assay enriches for DSB signal relative to the alkaline protocol, but “DSB-specific” overstates the case. Sensitivity is lower than the alkaline variant, and the protocol is technically unforgiving. “Halo” artifacts—diffuse chromatin coronas without directional migration—arise when supercoiled loops relax but lack the free ends required for electrophoretic movement. In PBMCs, where apoptotic debris is common, halos contaminate scoring unless strict viability gating (>85% trypan blue exclusion) is enforced before embedding [30].

For translational work, neither protocol alone suffices. A parallel design—alkaline for global burden, neutral for lethal DSB confirmation—is the minimum defensible approach, particularly in radiotherapy monitoring, where the clinical question is frank chromosomal breakage, not total lesion load.

### 2.2. Enzyme-Modified Comets: Unmasking Oxidative Damage

Standard SCGE is blind to intact oxidized bases. 8-oxoguanine, the canonical product of ROS attack, remains undetectable until converted into a strand break. This conversion requires exogenous glycosylases. Formamidopyrimidine DNA glycosylase (Fpg) is conventionally employed to reveal oxidized purines—primarily 8-oxoguanine and formamidopyrimidine adducts—while Endonuclease III (Nth) is used for oxidized pyrimidines such as thymine glycol. In practice, substrate specificities overlap, and neither enzyme is absolutely selective; the distinction is operational rather than mechanistic. Post-lysis incubation with these enzymes transforms cryptic oxidative lesions into scorable breaks [31,32].

The clinical utility is substantial. In autoimmune cohorts—RA, systemic lupus erythematosus (SLE), and systemic sclerosis (SS)—basal strand breaks may appear similar to those in controls, precisely because efficient in vivo repair clears physical damage within hours [33]. The underlying repair defect becomes apparent only when cells are challenged ex vivo: after ionizing radiation, lymphocytes from SLE and (Juvenile Rheumatoid Arthritis) JRA patients show 2–3-fold higher residual damage than controls at 30 min post-exposure, revealing delayed strand break rejoining [34]. Baseline comet assays thus capture a snapshot after repair has occurred, masking the functional deficiency that challenge assays expose. Fpg-sensitive sites, however, accumulate. They represent the oxidative “debt” of chronic inflammation, the molecular record of sustained ROS exposure that strand-break measurement alone cannot capture. In RA, Fpg-modified comet analysis revealed associations between oxidative lesion burden and polymorphisms in key BER genes—OGG1, MUTYH, UNG—that were invisible to standard alkaline protocols [12]. The enzyme-modified assay is frequently the only approach that separates patients from controls in cross-sectional designs where basal physical breaks have been cleared by constitutive repair.

Technical caveats apply. Enzyme activity is batch-dependent; each lot requires titration against a positive control (e.g., photosensitizer-treated cells). Incubation time matters: under-digestion leaves lesions unconverted; over-digestion introduces non-specific nicking. A buffer-only parallel slide is mandatory—the Fpg-sensitive signal is calculated by subtraction, and without this control, the data are uninterpretable [35,36,37,38].

### 2.3. Metrics, Throughput, and the Tail Length Fallacy

Legacy publications report Tail Length in micrometers. This metric is scientifically useless. Tail length scales with electrophoresis duration and voltage gradient; a 20 μm tail at 0.7 V/cm for 25 min cannot be compared to a 20 μm tail at 1.0 V/cm for 20 min [39]. Cross-study meta-analysis using tail length is an exercise in noise aggregation.

Acceptable metrics are two: % Tail DNA (fluorescence intensity in tail/total intensity) and Olive Tail Moment (product of tail length and Tail DNA fraction) [24]. Both are dimensionless or semi-normalized, and both correlate with lesion frequency in validation studies using ionizing radiation or defined H_2_O_2_ doses. The hComet COST Action (CA15132) guidelines—the closest approximation to an international consensus—mandate % Tail DNA as the primary endpoint for regulatory submissions [40].

Throughput remains the assay’s Achilles heel. Manual scoring of 50–100 cells per condition, replicated across treatment arms, demands hours of operator time and introduces scorer bias that inflates CVs beyond 30% [22,41,42]. Automated platforms (OpenComet, Comet Assay IV, HiComet) reduce this burden and improve reproducibility, but adoption in translational studies is inconsistent [43,44]. For any clinical trial with more than 50 subjects, automated scoring is a prerequisite for statistical defensibility [45].

### 2.4. From Damage Measurement to Repair Capacity: The Challenge Assay

Quantifying baseline damage answers the wrong question. The translational goal is not “how much damage exists now?” but “how efficiently can this patient’s cells repair a standardized insult?” The comet assay answers this through a challenge-recovery design: cells receive a defined genotoxic pulse (e.g., 50 μM H_2_O_2_, 7 μM tert-butyl hydroperoxide, 25 μM bleomycin, or 5 Gy ionizing radiation), then incubate in repair-permissive medium for defined intervals (typically 15, 30, 60, and 120 min) before lysis [31]. The slope of damage resolution—not the intercept—is the functional readout [46].

This kinetic approach distinguishes patients with elevated baseline damage (exposure history) from those with sluggish repair (pathway deficiency). The distinction matters clinically: a patient with high oxidative burden but intact repair may tolerate genotoxic therapy; one with equivalent burden but delayed kinetics may not [47,48].

The design has been validated in autoimmune cohorts. In rheumatoid arthritis and multiple sclerosis, PBMCs exhibit delayed resolution of oxidative lesions and DSBs compared to healthy controls, with repair efficiency correlating with disease status independently of baseline damage levels [12,46,49]. Subset-specific analysis of B cells, CD4^+^, and CD8^+^ T cells revealed divergent repair trajectories invisible to bulk PBMC measurements, opening the door to immunophenotype-specific DRC profiling [49].

**Figure 2:**
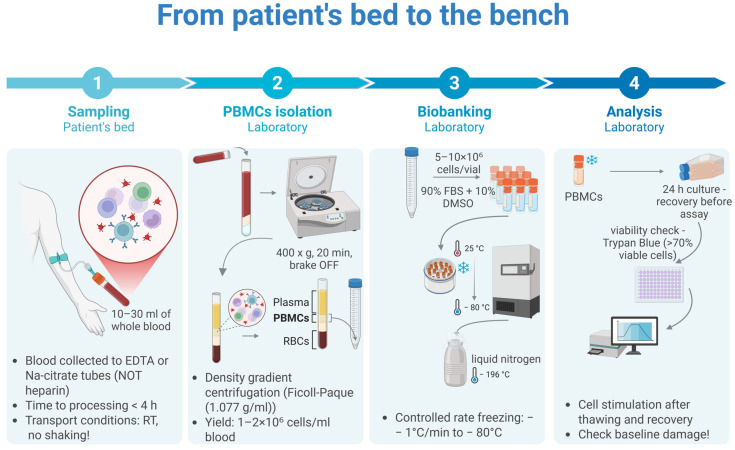
From bedside to bench: the standardized pre-analytical workflow. Schematic representation of the critical control points required to preserve DNA repair competence in biobanked PBMCs. (1) Sampling: Strict ischemia limit (<4 h) prevents proteomic degradation. (2) Isolation: Density gradient centrifugation must minimize granulocyte contamination. (3) Biobanking: Controlled-rate freezing (−1 °C/min) is mandatory to prevent intracellular ice crystal formation; direct transfer to −80 °C is prohibited. (4) Analysis: A post-thaw recovery period (culture in complete medium) allows for metabolic reactivation and ATP pool restoration. Viability assessment (Trypan Blue or Annexin V) with a threshold of >85% is required to exclude apoptotic “ghost” artifacts. Created in BioRender. Macieja, A. (2026) oe7w0mj.

Few published studies exploit this design despite its relevance. The reasons are logistical—time-course experiments multiply sample handling and require strict temperature control—but surmountable with appropriate workflow design (see Section 5, Figure 2). For biobanked samples, viability thresholds become critical: cryopreserved PBMCs with post-thaw viability below 85% yield artifactually elevated baseline damage and compressed dynamic range, potentially masking actual repair differences [18,50,51,52,53].

### 2.5. Quantification of Repair Kinetics: Metrics and Temporal Considerations

Quantification of repair kinetics lacks consensus [40]. Published studies report repair capacity using heterogeneous metrics: AUC, integration, half-time of repair (t½), residual damage at a fixed post-challenge time point, percent repair relative to initial damage, or rate constants derived from exponential decay fitting. Each approach carries assumptions—AUC weights all time points equally, t½ assumes first-order kinetics, and fixed-timepoint analysis discards trajectory information—and none have been adopted as a regulatory or consensus standard. This heterogeneity complicates cross-study comparison and effectively precludes meta-analysis [49,54,55,56].

Temporal windows must match the pathway under investigation. BER resolves the majority of oxidative lesions within 30–60 min; extending measurement beyond this window captures not BER efficiency but secondary processes—transcription-coupled repair, replication-associated damage tolerance, or apoptotic fragmentation [57,58]. Conversely, DSB repair via NHEJ exhibits biphasic kinetics: a fast component (complete within 1–2 h) representing simple breaks in euchromatin, and a slow component (4–24 h) reflecting complex lesions or heterochromatic DSBs requiring chromatin remodeling [59,60,61]. HR operates on still longer timescales and is restricted to S/G2 phase, rendering it largely inaccessible in quiescent PBMCs. A 60 min window appropriate for oxidative damage is inadequate for DSBs; a 120 min window captures fast-phase NHEJ but misses slow-phase resolution entirely [62].

A pragmatic solution: ordinal classification against control distribution. Rather than pursuing illusory precision with continuous metrics, DRC can be expressed as an ordinal variable anchored to the investigator’s own control population. Individual repair efficiency (percent damage resolved at the pathway-appropriate timepoint) is ranked against control quartiles: values above the median represent efficient repair, those in the second quartile marginally efficient, and those below the 25th percentile inefficient or absent repair. Quartile-based classification (preferred over tertiles for resolution, over quintiles for stability in moderate sample sizes) was chosen over parametric alternatives (z-scores, mixed models) because DRC distributions are typically right-skewed with outliers from hyper-damaged cells; rank-based categorization is distribution-free, robust to extreme values, and directly translatable to odds ratios without distributional assumptions. In autoimmune cohorts, this approach revealed odds ratios exceeding 40 for disease association in the lowest repair category—effect sizes obscured by reporting group means [12,63].

## 3. Surrogate Markers of Signaling: γH2AX and 53BP1 Analysis

The comet assay visualizes physical DNA fragmentation. Immunodetection of DDR proteins captures something different: the cellular reaction to damage. This distinction matters. A cell with defective Ataxia Telangiectasia Mutated (ATM) kinase may harbor frank DSBs yet mount no γH2AX response; conversely, replication stress can trigger robust H2AX phosphorylation without a single DSB. The two assays measure different biology and conflating them—treating γH2AX foci as equivalent to “breaks”—is a persistent source of confusion in the literature.

### 3.1. The Biology of γH2AX: Amplification and Its Consequences

Phosphorylation of histone variant H2AX at serine 139 (γH2AX) is catalyzed primarily by ATM in response to DSBs, with contributions from Ataxia Telangiectasia and Rad3-related (ATR; replication stress) and DNA-dependent Protein Kinase Catalytic Subunit (DNA-PKcs) (during NHEJ) [64]. A single DSB triggers phosphorylation of H2AX molecules across megabase chromatin domains, creating a microscopically visible “focus.” This amplification confers extraordinary sensitivity—single breaks are detectable—but sensitivity comes at the cost of specificity.

γH2AX is not a DSB counter [65]. It is a marker of chromatin remodeling in response to perceived damage. Conditions that activate ATM/ATR without frank breakage—stalled replication forks, R-loops, oxidative base damage, even transcriptional stress—produce γH2AX signal [64,66,67]. In proliferating cells, ATR-mediated phosphorylation during replication stress can exceed ATM-mediated signaling from frank DSBs; in quiescent PBMCs this contribution is minimal, but ex vivo stimulation or contamination with cycling progenitors reintroduces ATR-driven signal that inflates apparent damage independently of DSB burden [68]. In translational studies using PBMCs, this matters: circulating cells experience variable proliferative histories, oxidative environments, and pharmacological exposures, all of which modulate γH2AX independently of actual DSB burden.

### 3.2. Pathway Choice: The 53BP1/BRCA1 Antagonism

The utility of foci analysis extends beyond damage quantification to pathway discrimination. DSB repair proceeds through NHEJ, which is available throughout the cell cycle, and HR, which is restricted to S/G2 phases when a sister chromatid template is available [62]. The choice between pathways is not stochastic; it is actively regulated at the level of DNA end resection.

53BP1 (p53-binding protein 1) promotes NHEJ by shielding DNA ends from nucleolytic processing. It recruits effectors—RIF1, Shieldin complex—that physically block resection. In this configuration, breaks are repaired by direct ligation, error-prone but fast [69,70].

BRCA1, in complex with CtIP, antagonizes 53BP1. It promotes end resection, generating 3′ single-stranded DNA overhangs that RPA coats, then RAD51 Recombinase (RAD51). RAD51 nucleofilament formation marks commitment to HR—a slower, high-fidelity pathway requiring homologous template [71,72].

Immunofluorescence co-staining captures this competition: (a) 53BP1 foci indicate NHEJ-poised breaks and (b) RAD51 foci indicate HR commitment. The RAD51/γH2AX ratio in irradiated cells has emerged as a functional biomarker of HR competence (“HRD score”), used clinically to predict PARP inhibitor sensitivity in BRCA-mutant ovarian cancer [73,74].

In consequence, a critical caveat arises for PBMC studies. Resting lymphocytes reside in G0/G1. Without stimulation (Phytohemagglutinin (PHA), anti-CD3/CD28), they do not enter S-phase and cannot execute HR. RAD51 foci in unstimulated PBMCs are rare to absent regardless of BRCA status. The RAD51/γH2AX ratio, validated in cycling tumor cells, has limited applicability to quiescent clinical isolates. Investigators wishing to assess HR capacity in PBMCs must either: (a) stimulate cells into cycle before challenge, introducing proliferation-associated confounders, or (b) accept that they are measuring NHEJ-dominant repair in a G0/G1 population. Neither approach is ideal; both require explicit acknowledgment.

### 3.3. Imaging Approaches: Resolution, Throughput, and Their Trade-Offs

Immunofluorescence microscopy remains the reference method for foci analysis. Confocal imaging resolves individual foci, enables colocalization studies (e.g., γH2AX/53BP1 overlap), and captures spatial distribution within the nucleus. Foci at the nuclear periphery (heterochromatin-associated) behave differently from euchromatic foci; only imaging preserves this information [75,76].

Limitations are substantial. Manual scoring of 50–100 cells per condition is labor-intensive and prone to operator bias. “Pretty cell” selection—unconscious preference for well spread nuclei with unambiguous foci—inflates apparent damage in some samples and deflates it in others [77]. Inter-scorer variability exceeds 25% in multicenter studies [78,79].

High-content screening (HCS) platforms address throughput by automated image acquisition and algorithmic foci detection. It can process thousands of cells per sample [80]. However, automation introduces its own biases. Nuclear segmentation algorithms optimized for adherent cell lines perform poorly on PBMCs, which are small (6–10 μm), round, and prone to clumping [77]. Foci detection thresholds—intensity cutoffs, size minima, circularity parameters—are user-defined and rarely standardized. Two laboratories using identical samples but different HCS settings will report different foci counts. Algorithmic objectivity is not the same as accuracy.

For translational PBMC studies, it is essential to validate automated scoring against manual counts in a subset of samples. Report detection parameters explicitly. Consider reporting foci-positive cell fraction (binary: ≥N foci = positive) rather than mean foci per cell, as the former is more robust to segmentation errors.

## 4. Beyond the Microscope: NGS-Based Break Mapping

The methods described in Section 2 and Section 3—comet assay, γH2AX foci—quantify damage burden but remain genomically blind: they report how much breakage exists, not where it occurs. NGS technologies now map strand breaks at nucleotide resolution, revealing whether lesions cluster at fragile sites, promoters, or replication origins [81,82]. This precision has transformed mechanistic research. For translational applications, however, the picture is less favorable. NGS methods are included in this review not as clinical alternatives—they fail every practical criterion outlined below—but because they provide the orthogonal, nucleotide-resolution ground truth against which functional assays must ultimately be validated.

NGS-based break mapping is expensive, technically demanding, and slow—weeks from sample to data. This section examines the gap between mechanistic power and clinical utility, covering high-resolution break-mapping methods and the practical barriers that currently confine these approaches to research rather than routine monitoring.

A note on reporter assays: plasmid-based Host cell reactivation (HCR) offers pathway-specific repair quantification in cell lines but fails in primary lymphocytes. Resting PBMCs are refractory to transfection; electroporation achieves 10–30% efficiency, with substantial loss of viability, and the surviving transfected population represents a non-random subset. HCR has no practical role in PBMC-based DRC profiling [83,84].

### 4.1. Nucleotide-Resolution Break Mapping

Standard whole-genome sequencing (WGS) detects the consequences of DNA damage—mutations, structural variants, and the permanent scars of misrepaired lesions. It cannot detect transient breaks that are resolved before replication. Specialized break-mapping protocols fill this gap by ligating sequencing adapters directly to free DNA ends in situ, before cell lysis and fragmentation. Earlier methods, including Break-seq, established the principle of adapter ligation to free ends [85]; END-seq and BLISS represent current iterations with improved sensitivity and resolution [81,82]. END-seq, BLISS, and DSB Capture [86] share this principle but differ in sensitivity and input requirements. END-seq, the current gold standard, achieves nucleotide-resolution mapping but requires 2–10 million cells per sample—feasible for cell lines but challenging with limited clinical material [82]. BLISS operates on fixed cells or tissue sections with input counts as low as 10^5^ cells [86], thereby extending its applicability to archival samples. Linear amplification via in vitro transcription, rather than exponential PCR, enables recovery from minimal starting material while maintaining quantitative accuracy through unique molecular identifiers [81]. Both methods have transformed mechanistic research: revealing CRISPR-Cas9 off-target cleavage sites, mapping replication–transcription collision hotspots, and identifying recurrent fragile sites in cancer genomes. Unlike γH2AX ChIP-seq, which captures the megabase-scale chromatin domain phosphorylated around a break, these methods pinpoint the exact scission nucleotide. Recent extensions—sBLISS for suspension cells and organoids [87]—further extend applicability to patient-derived samples and archival biobank material, where fresh cell isolation is impractical.

### 4.2. Practical Barriers: Why NGS Remains a Research Tool

Despite their mechanistic power, NGS-based break-mapping methods face barriers that preclude routine clinical use. Turn-around time is prohibitive. Comet assay results are available within hours of sample collection [88], γH2AX Flow Cytometry within a day [89]. END-seq and BLISS require library preparation, deep sequencing, and bioinformatics analysis—a pipeline measured in weeks, not hours. The analysis itself is non-trivial: distinguishing proper biological breaks from handling-induced artifacts (ex vivo processing generates spurious free ends at mechanically fragile sites) demands specialized computational filtering and experienced interpretation [90].

Cost scales poorly. A single END-seq library is expensive, including sequencing; longitudinal monitoring—five timepoints across a treatment course—becomes fiscally untenable. By contrast, comet assays require reagents and labor, and Flow Cytometry adds the expense of antibodies, but neither approach NGS pricing.

Input requirements exclude common clinical scenarios. Most break-mapping protocols require 2–10 million cells for adequate library complexity. A standard blood draw yields 10–20 million PBMCs under optimal conditions. In lymphopenic patients—active SLE, post-chemotherapy, advanced HIV—yields drop to 1–5 million, insufficient for current protocols [91]. BLISS partially addresses this with lower input thresholds (~10^5^ cells), but even this remains demanding for serial sampling.

The verdict is clear: NGS-based break mapping serves as a powerful validation tool—confirming, for instance, that comet assay kinetics reflect actual DSB resolution rather than artifact—but it does not meet the scalability, speed, or cost requirements for frontline patient stratification or pharmacodynamic monitoring in clinical trials.

## 5. The Clinical Minefield: Pre-Analytical Variables and Confounders

Cell lines are metabolically predictable, patients are not. In translational cohorts—autoimmunity, oncology, chronic infection—the investigator confronts a sample that has been marinated in corticosteroids, methotrexate, or biologics; bathed in inflammatory cytokines; subjected to variable ischemia times between phlebotomy and processing; and, if biobanked, frozen and thawed under conditions that may or may not preserve repair capacity. This pharmacological and logistical noise routinely drowns the “signal” of DNA repair function. Most published studies ignore it. This is why functional biomarker research faces reproducibility issues.

**Figure 3:**
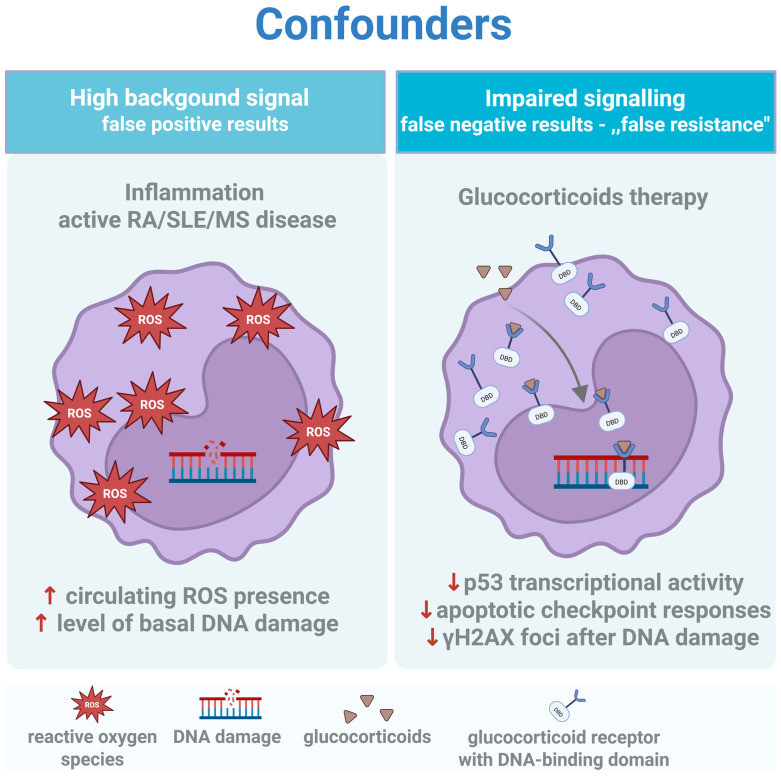
The confounder interactome: opposing biases in autoimmune cohorts. Systemic disease introduces two distinct forms of artifactual signal. (**Left)** (High Background): Chronic inflammation and circulating ROS create a “oxidative debt,” manifesting as elevated baseline strand breaks that mimic genomic instability. (**Right)** (Impaired Signaling): Glucocorticoid therapy (exogenous steroids) suppresses p53-dependent transcriptional activity and apoptotic checkpoints. This results in blunted H2AX foci formation and reduced cell death after challenge, creating a phenotype of “false resistance” where cells fail to signal damage despite its presence. The arrows ↑ and ↓ indicate the increase and decrease of the indicated parameters, respectively. Created in BioRender. Macieja, A. (2026) https://BioRender.com/eo3v6e0.

**Figure 4:**
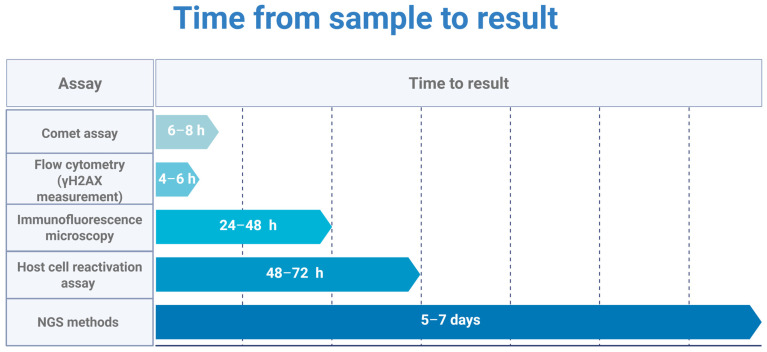
Benchmarking Turn-around Times: Clinical feasibility vs. resolution. Comparison of the minimum “sample-to-result” interval for primary DNA repair assays. Comet assay and Flow Cytometry provide data within a clinically relevant window (<24 h), enabling their use for pharmacodynamic monitoring or acute patient stratification. Host cell reactivation (HCR) and NGS-based methods (e.g., END-seq) require multi-day processing pipelines (48 h to 7 days), limiting their utility to retrospective mechanistic analysis rather than real-time clinical decision-making. Created in BioRender. Macieja, A. (2026) https://BioRender.com/nkfwg6i.

This section dissects two categories of confounders. The first is biological: how systemic inflammation and immunosuppressive therapy alter DDR independently of any underlying repair defect (Section 5.1; see Figure 3 and Table 1). The second is logistical: how sample collection, processing delays, and cryopreservation introduce artifacts that masquerade as impaired repair (Section 5.2; see Figure 2 and Figure 4). Both must be controlled—or at a minimum, documented—for DRC data to be interpretable.

### 5.1. Pharmacological and Inflammatory Confounders

In autoimmune disease, the DNA damage response operates within a web of competing influences—what might be termed a “confounder interactome” (Figure 3). Two forces dominate: chronic oxidative stress from the disease itself [92], and immunosuppressive therapy intended to control it. Both distort DRC measurements in predictable but frequently ignored ways [17,93,94].

Patients with active disease—SLE flare, RA exacerbation, MS relapse—exhibit elevated circulating reactive oxygen species [17,95,96]. In the comet assay, this manifests as severe basal damage at time zero, before any ex vivo challenge. The unwary investigator interprets this as “genomic instability” or “repair deficiency.” Often, it is neither. It is oxidative saturation: the BER machinery is functional but overwhelmed by substrate. Repair kinetics may be entirely normal once the oxidative burden is accounted for. The solution is methodological: Fpg-modified comet assays (Section 2.2) distinguish accumulated oxidative lesions from physical strand breaks, and challenge-recovery designs (Section 2.4) separate baseline burden from repair capacity. Without these controls, inflammatory cohorts are systematically misclassified.

Corticosteroids are near-universal in autoimmune management, yet their impact on DNA damage response signaling is rarely controlled for in functional assays. Glucocorticoids are known to interfere with p53-dependent transcriptional programs through direct GR–p53 crosstalk and context-dependent modulation of the MDM2–p53 axis, resulting in attenuation of DNA damage-induced checkpoint and apoptotic signaling [97,98]. In γH2AX assays, this produces a paradox: steroid-treated patients show fewer foci after challenge, not because they repair efficiently, but because they fail to signal. The damage exists; the cellular alarm does not sound. This “false resistance” phenotype has led to erroneous conclusions about repair capacity in multiple published cohorts.

The problem extends beyond steroids. Methotrexate depletes folate pools, altering nucleotide availability for repair synthesis [99]. NSAIDs inhibit cyclooxygenase-mediated prostaglandin signaling, which cross-talks with ATM/ATR pathways through mechanisms still being elucidated [100]. Biologics targeting TNF-α or IL-6 reduce inflammatory ROS—potentially improving apparent DRC by lowering baseline damage—confounding longitudinal comparisons before and after treatment initiation [101].

Cohort stratification by drug exposure is mandatory, not optional. At minimum, investigators must document: (1) corticosteroid dose and duration (distinguish pulse therapy from chronic low-dose from no exposure); (2) DMARD status (MTX, sulfasalazine, leflunomide); (3) biologic exposure; (4) NSAID use in the 48 h preceding sampling. Ideally, blood collection should occur at a standardized interval relative to steroid dosing—trough levels (immediately before morning dose) minimize acute pharmacodynamic effects. Table 2 summarizes common drugs, their known DDR interactions, and recommended stratification approaches.

### 5.2. Biobanking for Functional Assays: From Passive Storage to DDR Fidelity

Standard biobanking protocols optimize for molecular analytes. DNA is stable; with care, RNA survives; serum and plasma tolerate moderate variations in handling. Functional assays demand more. The DNA damage response is not a molecule to be extracted—it is living enzymatic machinery. A PBMC sample may pass viability screening (membrane intact, trypan blue excluded) yet harbor inactivated ATM kinase, degraded MRN complex, or depleted ATP pools. Such samples are “viable but incompetent”—they will yield data, but the data will be wrong. Biobanking for DRC assessment requires pharmaceutical-grade rigor, not passive specimen storage.

Viability dyes report membrane integrity, nothing more. A cell with an intact plasma membrane but proteolytically degraded DDR machinery will exclude trypan blue and produce a comet—but its repair kinetics will be artifactually slow. This “functional senescence” is invisible to standard quality control (QC). It accumulates with every hour of ischemia, every suboptimal freeze–thaw cycle, every protocol deviation. The only defense is process control: standardize ruthlessly, document obsessively, verify functionally.

Time from phlebotomy to cryopreservation determines functional preservation. Whole blood at room temperature is not inert—granulocytes activate, releasing reactive oxygen species and proteases that inflict bystander damage on lymphocytes. The defensible threshold is 4 h; beyond 6 h, baseline comet damage rises detectably; beyond 12 h, samples show elevated strand breaks and blunted response to ex vivo challenge [33]—not biological resistance, but proteomic exhaustion [102]. For multicenter studies where same-day processing is impossible, on-site separation using CPT tubes (citrate-polymer gel barrier) immediately after collection is preferable to shipping whole blood overnight.

Dimethyl Sulfoxide (DMSO; 10% final concentration) is the standard cryoprotectant—and a time-dependent genotoxin. At room temperature, DMSO induces oxidative damage within minutes. The protocol must reflect this: add DMSO to pre-chilled cell suspension (4 °C), mix rapidly, transfer immediately to controlled-rate freezing. Every minute at room temperature degrades the sample [103].

Rapid freezing forms intracellular ice crystals that shear chromatin and rupture nuclear architecture. Slow freezing (−1 °C per minute) permits osmotic dehydration, allowing water to exit cells before crystallization. Isopropanol-bath containers (Mr. Frosty) achieve this rate passively; automated controlled-rate freezers offer precision and audit trails. Direct transfer to −80 °C without rate control is unacceptable for functional assays.

Process standardization is necessary but insufficient. Freezer failures and temperature excursions occur. The solution is prospective functional validation: every freezing batch includes “sentinel” aliquots from a characterized healthy donor. Sentinels are thawed at defined intervals (6 months, 12 months, annually thereafter) and subjected to the same DRC assay applied to patient samples. If sentinel repair capacity degrades beyond pre-specified limits (e.g., >20% decline in percent recovery after challenge), the entire batch is flagged. This is pharmaceutical-grade quality control. Biobanks that cannot demonstrate sentinel stability cannot claim functional fidelity.

For biobanked samples used in DRC studies, record and report: (1) ischemia time; (2) processing method (Ficoll, CPT, other); (3) cryoprotectant concentration and temperature at addition; (4) freezing method and rate; (5) storage temperature and duration; (6) temperature excursions; and (7) post-thaw viability. Studies lacking this documentation cannot be evaluated for pre-analytical confounding.

### 5.3. Post-Thaw Handling: The Recovery Imperative and the “Ghost” Artifact

A correctly frozen sample can still yield unusable data if thawed incorrectly. The post-thaw window—from liquid nitrogen to assay—introduces its own artifacts, distinct from pre-freeze errors but equally destructive.

Cryopreservation induces chromatin compaction and metabolic arrest [104]. Cells thawed and immediately challenged show elevated baseline damage and compressed repair kinetics [105]—not because repair is deficient, but because the machinery has not yet reactivated. A recovery period of 2–4 h in complete medium at 37 °C is mandatory. This allows the restoration of ATP pools, the re-establishment of chromatin accessibility, and the normalization of enzymatic activity. Studies omitting this step systematically overestimate repair deficiency. The difference between “impaired DRC” and “metabolic shock” is two hours of incubation.

Standard thaw protocols call for rapid dilution and washing, but trace amounts of cryoprotectant persist. At concentrations above 0.1%, DMSO interferes with enzyme-modified comet assays—Fpg activity is particularly solvent-sensitive. Validate removal by protocol or report residual concentration. This is not pedantry; it is the difference between “oxidative repair defect” and “assay interference.”

Apoptotic cells produce characteristic “ghost” comets—tiny heads with diffuse, disconnected tails spanning the microscope field. These are not hyper-damaged cells; they are dead cells undergoing caspase-mediated DNA fragmentation. Their inclusion inflates damage scores and compresses apparent repair curves. The problem is insidious: ghosts arise from cells that were viable at freezing but died during storage or thaw. A sample frozen at 95% viability may thaw at 80%, and that 15% difference contaminates every measurement.

Viability assessment must accompany every functional assay. Trypan blue exclusion is the minimum; it detects membrane rupture but misses early apoptosis. For higher stringency, Annexin V/PI dual staining identifies cells that are membrane-intact but phosphatidylserine-positive—committed to death, functionally useless, and invisible to trypan blue. Strict threshold: reject samples with viability below 85% by either method.

Fresh versus frozen is not equivalent. Even optimally processed cryopreserved PBMCs show higher baseline damage and marginally slower repair kinetics than fresh isolates from the same donor. The difference is slight but reproducible, likely reflecting irreversible chromatin reorganization during freeze–thaw. For cross-sectional studies (patients vs. controls), this is acceptable—both groups experience the same artifact. For longitudinal studies comparing fresh baseline samples to frozen follow-ups, the artifact confounds the biology. Protocol consistency is mandatory: all samples fresh, or all samples frozen. Mixing is not acceptable.

These are not recommendations; they are requirements for interpretable data: (1) Recovery period: minimum 2 h at 37 °C before challenge. (2) Viability: reject samples <85%. (3) Residual DMSO: confirm removal or document concentration. (4) Fresh/frozen consistency: never mix within a longitudinal comparison. (5) Ghost exclusion: train scorers explicitly or use automated systems with size/morphology gating.

## 6. Towards Standardization: Validation, Reporting, and Regulatory Compliance

The preceding sections cataloged what can go wrong: pharmacological confounders that masquerade as repair phenotypes, handling artifacts that mimic genomic instability, and metric heterogeneity that precludes cross-study comparison. The litany of problems raises an obvious question: can functional DRC profiling ever achieve clinical utility, or is it condemned to remain a research curiosity?

The answer depends on standardization. Validated clinical assays—quantitative PCR for viral load, immunoassays for cardiac troponin, Flow Cytometry for CD4 counts—achieve inter-laboratory CVs below 15% because protocols, reagents, and reporting are harmonized. Functional repair assays currently operate as artisanal methods: each laboratory uses its own comet scoring parameters, its own γH2AX antibody clone, its own definition of “efficient repair.” This fragmentation is why meta-analysis fails and why regulators remain skeptical.

This section addresses the path forward. We first benchmark the performance of existing assays—sensitivity, specificity, throughput, and reproducibility (Table 1). We then examine the regulatory requirements for clinical translation under the ISO 15189 and CLIA frameworks, identifying why most research assays fail to qualify. Finally, we propose a Minimum Reporting Standard for DRC studies (Table 3)—not a rigid protocol, but a documentation floor below which data cannot be meaningfully interpreted or compared.

### 6.1. The Variability Crisis and the hComet Response

Functional DRC assays suffer from reproducibility problems that would disqualify them from clinical use under current regulatory standards. Table 1 summarizes the performance characteristics of primary methods—comet assay variants, γH2AX foci, Flow Cytometry—across published validation studies. Inter-laboratory coefficients of variation for the same samples routinely exceed 30%, sometimes approaching 50%. For comparison, validated clinical assays (HbA1c, cardiac troponin, CD4 counts) maintain CVs below 10–15%. The gap is not subtle [29,42,106].

The hComet COST Action (CA15132) conducted the most systematic analysis of comet assay reproducibility to date. Ring trials across European laboratories identified multiple drivers of inter-center divergence: electrophoresis tank geometry and buffer depth; voltage gradients (V/cm varies with tank design and power supply); agarose concentration and lot; lysis duration and temperature; unwinding time in alkaline buffer; and—critically—scoring parameters (software settings, operator training, threshold definitions). Each variable contributes; their interactions multiply. A protocol that works in Warsaw may not transfer to Madrid without recalibration.

The ring trials yielded one robust finding: while absolute % Tail DNA values diverged substantially across laboratories, the relative ranking of samples remained consistent. A sample scored as “high damage” in one center was high in all centers; “low” remained low. This suggests that the assay measures something real, but that the scale is laboratory-specific—akin to temperature measured in Celsius versus Fahrenheit. The implication is both reassuring and limiting. Within-study comparisons (patient vs. control, pre- vs. post-treatment) are defensible. Cross-study comparisons using absolute values from different laboratories are not.

The problem is not unique to SCGE. γH2AX immunofluorescence shows comparable heterogeneity: foci counts depend on antibody clone, fixation protocol, permeabilization stringency, microscope settings, and—for automated scoring—algorithm parameters [107]. Flow cytometric γH2AX suffers from MFI drift across cytometers and sessions. Published studies rarely report sufficient methodological detail to assess comparability. The field lacks not only standardized protocols but also standardized reporting of protocols.

If absolute values are laboratory-specific, normalization is mandatory. Every experimental run must include an Internal Quality Control (IQC): cryopreserved aliquots from a characterized reference population—typically PBMCs from healthy donors, optionally including an irradiated “high damage” standard. Patient results are then expressed relative to IQC performance in that run:

Normalized Damage Index = (Patient value − IQC baseline)/(IQC challenged − IQC baseline)

This approach corrects for batch-to-batch technical drift—voltage fluctuations, reagent lot changes, scorer variability—by anchoring every dataset to an internal reference. It does not solve inter-laboratory comparability (each lab’s IQC is different), but it ensures that longitudinal data within a study are interpretable. For multicenter trials, a shared IQC distributed from a central biobank can extend this normalization across sites.

A critical distinction: inter-laboratory variability is noise; inter-individual variability is signal. Healthy donors exhibit substantial person-to-person differences in baseline DRC—up to 2-fold variation in repair kinetics within age-matched cohorts has been reported, reflecting polymorphisms in repair genes, lifestyle factors, and occult inflammatory burden. This biological heterogeneity is not an artifact to be eliminated; it is the substrate from which disease-associated deficits must be distinguished. IQC normalization corrects for technical drift but preserves inter-individual differences. The implication for study design is clear: control populations must be sufficiently large (*n* ≥ 30) to characterize the normal distribution, and patient values should be interpreted against this distribution rather than against a single “reference” donor whose DRC may itself be atypical.

Table 1 compares assay performance: sensitivity (minimum detectable damage), specificity (DSB vs. total lesions vs. oxidative), throughput (samples per operator-day), and reported inter-laboratory CV from published ring trials or validation studies.

### 6.2. Regulatory Hurdles: The Absence of Certified Reference Materials

Clinical laboratory accreditation under ISO 15189 or CLIA requires traceability: every measurement must be calibrated against a reference of known value, and that reference must itself be traceable to a higher-order standard—ultimately, to SI units or internationally recognized Certified Reference Materials (CRMs) [108]. For hemoglobin, such standards exist [109]. For cardiac troponin, they exist. For DNA damage, they do not.

The problem is not bureaucratic indifference; it is technical intractability. A CRM for strand breaks would need to be a biological matrix—cells or isolated DNA—with a defined, stable, and reproducible frequency of lesions. But DNA damage is inherently dynamic. Strand breaks in solution undergo spontaneous religation or degradation. Oxidative lesions are chemically labile. Cells carrying defined damage die, repair, or accumulate secondary lesions during storage [110]. There is no equivalent of a “standard glucose solution” for genotoxicity: no bottle of damaged DNA with a certificate stating “47.3 ± 2.1 breaks per 10^9^ base pairs.”

IQC (Section 6.1) addresses within-laboratory drift—they anchor each experimental run to a reference sample processed in parallel. But IQCs are laboratory-specific; they do not enable inter-laboratory comparison because each site uses different donor material, different damage induction protocols, and different storage conditions. CRMs would solve this: identical aliquots distributed globally, with certified damage values established by a reference laboratory using validated methods. Proficiency testing—where participating laboratories analyze blinded CRM samples—would then quantify inter-site variability and identify outliers. Without CRMs, proficiency testing is impossible, and regulatory bodies have no basis for accrediting DRC assays [106].

Current substitutes and their limitations. Most laboratories rely on “home-brew” controls: cells treated with hydrogen peroxide, ionizing radiation, or chemical mutagens at defined doses. These serve as positive controls but not as CRMs. Damage induction is dose-dependent but not precisely quantified at the lesion level; batch-to-batch variability is substantial; storage stability is poorly characterized. Irradiated cell pellets can be cryopreserved and shared, but without orthogonal validation of the actual break frequency, they remain arbitrary references—useful for QC trending but inadequate for calibration.

Accurate reference materials for DRC assays would require: (1) a stable matrix—likely lyophilized cells or fixed nuclei—that survives shipping and long-term storage without lesion loss or gain; (2) orthogonal quantification of damage by chemistry-based methods (e.g., LC-MS/MS for 8-oxoguanine, qPCR-based lesion frequency assays for strand breaks) rather than the functional assay being calibrated; (3) multi-center validation demonstrating that participating laboratories recover the certified value within acceptable tolerance; (4) ongoing stability monitoring. This is a substantial undertaking—years of development, significant funding, and coordination across regulatory and academic bodies. No such initiative currently exists at the scale required.

Until CRMs are available, functional DRC data cannot meet the evidentiary standards required for FDA or EMA approval as companion diagnostics [111]. Translational studies can proceed—and should—but claims must be framed appropriately. Relative comparisons (patient vs. matched control, pre- vs. post-treatment) are defensible when rigorous IQC is employed. Absolute claims (“this patient’s repair capacity is 47% of normal”) are not, because “normal” has no certified definition. Every publication should explicitly state this limitation rather than leaving it to regulators to discover during submission review.

### 6.3. A Minimum Reporting Standard for DRC Studies

The preceding sections documented how undisclosed variables—such as ischemia time, steroid exposure, scoring parameters, and viability thresholds—transform interpretable biology into methodological noise. The solution is not more sophisticated statistics; it is enforced transparency. Suppose reviewers and readers cannot determine whether a study controlled for known confounders; they cannot evaluate its conclusions. The field needs a reporting floor.

We propose a “Minimum Information for DNA Repair Profiling” (MIDRP) checklist, modeled on the MIAME standard that transformed microarray reproducibility [112]. MIDRP does not prescribe protocols—laboratories may use different comet variants, different γH2AX antibodies, and different scoring systems. What it mandates is disclosure: explicit documentation of variables known to affect outcome, reported in sufficient detail for replication or critical evaluation [113]. Table 3 presents the complete checklist; the rationale for each element derives from evidence reviewed in Section 2, Section 3, Section 4 and Section 5.

Sample-handling artifacts masquerade as biology (Section 5.2 and Section 5.3). MIDRP requires reporting of: (1) ischemia time—interval from phlebotomy to processing, with explicit statement if exceeding 4 h; (2) processing method—Ficoll, CPT, or other, with centrifugation parameters; (3) cryopreservation protocol—DMSO concentration, cooling rate, storage temperature, and duration; (4) post-thaw recovery period—duration and conditions before assay; (5) viability assessment—method (trypan blue, Annexin V/PI) and threshold applied (minimum 85%) [28]; (6) fresh versus frozen status, with explicit statement that sample types were not mixed within longitudinal comparisons.

Protocol heterogeneity drives inter-laboratory variability (Section 6.1). MIDRP requires: (1) assay variant—alkaline comet, neutral comet, enzyme-modified (specify enzyme, concentration, incubation time), γH2AX IF, γH2AX flow; (2) damage metric—% Tail DNA, Olive Tail Moment, foci per cell, MFI, with explicit justification for choice; (3) scoring method—manual versus automated, software name and version, detection parameters (thresholds, size gates); (4) cells scored per sample—minimum and actual; (5) positive and negative controls—what was used, how prepared, whether included in every run [48]; (6) Internal Quality Control—IQC material, normalization formula if applied; (7) blinding—whether scorers were blinded to sample identity.

Challenge-recovery designs introduce additional variables (Section 2.4). MIDRP requires: (1) challenge agent—compound, concentration, exposure duration, temperature; (2) recovery timepoints—all timepoints sampled, with rationale for selection based on pathway kinetics; (3) repair metric—AUC, t½, residual damage, percent recovery, or ordinal classification against control distribution; (4) baseline handling—whether time zero represents pre-challenge or immediate post-challenge.

Pharmacological confounders alter DDR independently of repair capacity (Section 5.1). MIDRP requires: (1) corticosteroid exposure—drug, dose, duration, timing of last dose relative to sampling; (2) DMARD and biologic exposure—current medications at time of sampling; (3) NSAID use—within 48 h of sampling; (4) disease activity—standardized score (DAS28, SLEDAI, EDSS) at time of sampling; (5) cell cycle status—if assessed, method and distribution.

We recommend that journals require MIDRP compliance as a condition of publication (Table 3). Incomplete disclosure should not trigger automatic rejection—clinical realities sometimes preclude complete documentation—but omissions must be explicitly acknowledged, and their potential impact on interpretation discussed. The goal is not bureaucratic box-checking; it is a culture shift toward transparency. Studies that cannot document basic handling parameters cannot claim clinical relevance.

Adherence to MIDRP does not guarantee scientific truth. A perfectly documented study may still reach wrong conclusions. But it ensures that peer reviewers, replicators, and clinicians can distinguish biological signal from methodological artifact—and that the field can learn from failures rather than endlessly repeating them.

## 7. Conclusions and Future Perspectives

The stratification of patients for DNA-damaging therapy has relied on a static proxy: the genomic sequence. A BRCA1 mutation predicts HR deficiency; a wild-type sequence implies competence. This paradigm is incomplete. Mutations do not guarantee functional defects—epigenetic silencing, protein mislocalization, and post-translational dysregulation can inactivate repair in the absence of any coding variant [114]. Conversely, a wild-type genotype does not ensure proteomic competence—a cell under oxidative siege or pharmacological suppression may harbor intact genes but crippled machinery. The gap between genotype and phenotype is where patients are lost.

Functional DNA repair profiling closes this gap. The comet assay, γH2AX quantification, and kinetic challenge-recovery designs measure what genomics can only infer: the actual capacity of a patient’s cells to resolve damage. The technology exists. What does not exist—yet—is the standardization required to translate research observations into clinical decisions.

This review has cataloged the following obstacles: pharmacological confounders that masquerade as repair phenotypes, sample handling artifacts that mimic genomic instability, metric heterogeneity that precludes cross-study comparison, and the absence of Certified Reference Materials that block regulatory approval. None of these obstacles is insurmountable. Each has a solution—or at least a mitigation—described in the preceding sections. The question is whether the field will implement them.

The end of artisanal methodology. Functional DRC assays have operated as craft methods: each laboratory developing its own protocols, its own scoring criteria, its own internal standards. This heterogeneity was tolerable when the goal was mechanistic insight. It is not tolerable when the goal is clinical deployment. Industrial rigor—the standardization that transformed PCR from a research curiosity into a diagnostic backbone—must now be applied to repair profiling. The MIDRP framework (Table 3) represents a first step: not a protocol mandate, but a transparency requirement that enables evaluation and replication. Journals should adopt it. Funding agencies should require it. Laboratories that cannot document their pre-analytical handling should not claim clinical relevance.

Automation is necessary but not sufficient. Automated liquid handling for comet assays and high-content screening for foci analysis will reduce operator variability—a genuine advance. AI-driven image analysis may improve scoring consistency and ghost/artifact discrimination. But automation addresses only one source of error. An ideally automated assay applied to a poorly handled sample will produce reproducible garbage. The “bedside-to-bench” workflow—from phlebotomy through cryopreservation to assay—demands equal attention. Sample logistics are not ancillary to the science; they are the science when the readout is functional rather than molecular.

A note on validation. The kinetic challenge-recovery approach advocated in this review is not theoretical. Our group has applied it to autoimmune cohorts—RA and MS —and has demonstrated that repair kinetics, expressed as AUC or ordinal classification against control quartiles, distinguishes patients from controls, with effect sizes that static baseline measurements cannot achieve. Subset-specific analysis of B cells, CD4^+^, and CD8^+^ T cells reveals differences in repair trajectories that are invisible to bulk PBMC measurements. These are proof-of-concept data, not definitive validation—but they demonstrate that standardized functional profiling is achievable in real clinical populations, not only in cell lines.

The path forward. Functional DRC assays will not replace genomic testing; they will complement it. A patient with a BRCA1 mutation and intact repair kinetics is biologically different from one with the same mutation and sluggish kinetics—and may respond differently to PARP inhibition. The combination of genotype and functional phenotype offers precision that neither alone can provide. Realizing this potential requires coordinated action:

- For researchers: We recommend adopting challenge-recovery designs over static measurements, reporting all pre-analytical variables, and employing Internal Quality Controls with appropriate normalization. Manuscripts should provide sufficient methodological detail to enable replication—undisclosed critical methods undermine the field’s credibility and slow clinical translation.
- For journals: We encourage requiring MIDRP-compliant reporting as a condition of publication. Manuscripts claiming clinical relevance should document sample handling to a standard that allows critical evaluation of potential confounders.
- For biobanks: Functional assays demand different quality standards than molecular extraction. We recommend implementing sentinel vial QC protocols and documenting ischemia thresholds as part of routine biobanking practice.
- For regulators: Investment in Certified Reference Material development is essential. Without CRMs, DRC assays cannot achieve the evidentiary standard required for companion diagnostic approval—a gap that currently blocks clinical translation.

The tools for functional DNA repair profiling are mature. The clinical need is acute—patients receiving genotoxic therapy without knowledge of their repair capacity are treated empirically, and some pay for that empiricism with toxicity or resistance. What remains is methodological discipline: the willingness to treat sample handling with the same rigor as molecular analysis, and to demand transparency where opacity has been tolerated. The transition from artisanal assay to clinical standard is not a technical problem. It is a cultural one. The field must decide whether functional repair profiling will mature into clinical utility or remain a perpetual research curiosity, always promising, never delivering.

## Figures and Tables

**Table 1:** Quantitative benchmarking of functional DNA repair assays for clinical translation.

Method/Assay	Target Lesion and Resolution	LOD (Sensitivity Limit)	Input Material (Cells/Sample)	Throughput (Samples/Day)	Cost Est. (USD/Sample)	Regulatory Status (QC Feasibility)	Critical Technical Limitation
Alkaline Comet Assay	SSBs, DSBs, Alkali-labile sites. Single-cell resol.	~0.05 Gy equation (~50 breaks/cell).	Low. ~10,000 cells (ideal for scarce samples).	20–40 (Manual)100+ (Automated)	USD 15–25 (Labor-intensive)	RUO. High inter-lab CV (>30%). Requires internal calibration standards (hComet).	Specificity. Cannot distinguish SSBs from DSBs without enzyme modification. “S-phase ghosts” mimic damage.
Neutral Comet Assay	DSBs primarily. Single-cell resol.	~1–2 Gy equation (Lower sensitivity than alkaline).	Low. ~10,000 cells.	20–40	USD 15–25	RUO. Lacks standardized lysis buffers compared to alkaline (OECD 489).	Quantification. Halo/Tail boundaries are often diffuse; high operator bias in manual scoring.
γH2AX Foci (Microscopy)	DSB Signaling (p-Ser139). Spatiotemporal foci.	1 Focus ≈ 1 DSB. Ultimate sensitivity.	Medium. 50,000–100,000 (Cytospin loss).	10–20 (Confocal)200+ (High-Content Screening)	USD 40–60 (Antibodies + Imaging time)	Validatable. ISO 15189 compliance possible with automated counting algorithms.	Indirect Readout. Measures chromatin remodeling, not physical breaks. Pan-staining in apoptosis/S-phase.
γH2AX (Flow Cytometry)	DSB Signaling (Population MFI).	Global Shift. Requires >10% shift in MFI vs. control.	Medium. 100,000+ (Required for gating).	High. >200 (96-well plate format).	USD 10–20 (Efficient for cohorts).	High Potential. Standardized beads allow instrument cross-calibration.	No Spatial Resolution. Cannot distinguish 10 cells with 1 focus vs. 1 cell with 10 foci.
Plasmid Reporters (e.g., HCR)	Specific pathway kinetics (NHEJ/HR).	High. Dependent on transfection efficiency.	N/A for PBMCs. Requires transfection (poor in primary cells).	Low/Medium.	USD 50+ (Transfection reagents).	Basic Research Only. Artificial plasmid substrates do not reflect chromatin context.	Clinical Incompatibility. Not feasible for routine PBMC screening without viral transduction (safety risks).
NGS Mapping (e.g., END-seq)	Physical DSB ends at nucleotide resolution.	Base-pair resolution. Maps exact genomic location.	Very High. 1–5 million cells required.	Very Low. TAT: 2–3 weeks.	>USD 500 (Library prep + Sequencing).	RUO. Bioinformatics complexity and cost prohibit routine diagnostic use.	“Scars vs. Breaks”. Standard WGS sees mutations; specialized Break-seq is too complex for routine use.

Comparative analysis of assay performance metrics. Cost estimates reflect reagent and labor overheads (excluding capital equipment amortization). Throughput estimates assume a standard academic workflow (single operator). Cost estimates include labor hours, which represent the primary expense in manual assays like the comet assay. Abbreviations: LOD—Limit of Detection; TAT—Turn-around Time; RUO—Research Use Only; ISO—International Organization for Standardization.

**Table 2:** The translational matrix: mapping DNA repair readouts to drug classes and clinical phenotypes.

Clinical Context/Drug Class	Mechanism of Action (MoA)	Recommended Assay Config.	Diagnostic Signature (Expected Readout)	Critical Interpretation Pitfall
Glucocorticoids (e.g., Prednisone, Dexamethasone)	Anti-inflammatory; Modulation of apoptotic thresholds and p53 signaling.	Flow Cytometry (γH2AX) + Annexin V.	Reduced basal γH2AX intensity; Blunted response to IR challenge.	False Resistance. Lower signaling may mimic “efficient repair” but actually reflects suppressed DDR signaling or elimination of damaged cells via silent apoptosis.
Alkylating Agents (e.g., Cyclophosphamide, Cisplatin)	DNA Crosslinking (Interstrand/Intrastrand); Stall replication forks.	Modified Comet Assay (Retardation assay).	Reduced Tail Moment. Crosslinks prevent DNA migration, creating “smaller” comets than control.	The “Zero Damage” Illusion. Standard alkaline comet will show less damage (shorter tails) despite lethal genotoxicity. Must compare to H_2_O_2_-treated control to see migration inhibition.
Topoisomerase II Inhibitors (e.g., Etoposide, Doxorubicin)	Stabilizes cleavable complex (DSB formation).	Neutral Comet or TARDIS (Trapped in Agarose DNA Immunostaining).	Rapid formation of DSB “tails” or trapped protein-DNA complexes.	Reversibility. Topo-lesions are rapidly reversed upon drug washout. Timing is critical (measure at peak concentration, not 24 h later).
PARP Inhibitors (e.g., Olaparib)	Inhibition of SSB repair → DSB accumulation in HR-deficient cells (Syntethic Lethality).	γH2AX/RAD51 Foci (Dual Staining).	High γH2AX + Low RAD51 = HR Deficiency (Responder).	Proliferation Bias. PARPi toxicity is replication-dependent. Non-cycling PBMCs (resting) will show minimal signal regardless of HR status. Requires ex vivo stimulation.
Chronic Inflammation (SLE, RA, Sepsis)	ROS-induced oxidative stress (8-oxoG accumulation).	Alkaline Comet + Fpg enzyme.	Significant tail increase only after Fpg digestion (oxidative lesions) vs. buffer control.	SSB vs. DSB Confusion. High alkaline comet signal often misinterpreted as “breaks”. Without Neutral Comet confirmation, this is usually just base damage/alkali-labile sites, not frank DSBs.

**Table 3:** The MIDRP checklist (Minimum Information for DNA Repair Profiling). To ensure reproducibility and distinguish biological variance from technical artifacts, the following parameters must be reported in translational studies.

Category/Domain	Reporting Requirement (Checklist)
A. Pre-Analytical Variables (Sample Provenance)	Ischemia Time: Duration from blood draw to processing (Target: <4 h). Processing Temp: Room Temp vs. 4 °C. Cryopreservation: Medium (e.g., 10% DMSO) and Cooling Rate (−1 °C/min). Post-Thaw Viability: Threshold used (Recomm.: >85%). Recovery Period: Duration of “resting” post-thaw (Recomm.: 2–4 h).
B. Clinical Confounders (The Meta-Data)	Steroid Load: Dosage (mg/day) and type of corticotherapy. Sampling Timing: Relative to drug administration (Peak vs. Trough). Inflammation: CRP/PCT levels (to exclude acute infection).
C. Assay Specifications (Technical Rigor)	Comet Assay: Voltage Gradient (V/cm) and Amperage. Metric: % Tail DNA or OTM (Reject: Tail Length). Cell count: >50 cells/replicate. γH2AX/Foci: Definition of “Focus” (Threshold algorithm). Gating Strategy: Exclusion of S-phase/Apoptosis.
D. QC and Statistics	Internal Standard: Inclusion of Calibration Control in every run. Blinding: Masking of clinical status during scoring. Raw Data: Availability of raw images/FCS files.

## Data Availability

No new data were created or analyzed in this study. Data sharing does not apply to this article.

## Abbreviations

The following abbreviations are used in this manuscript:

53BP1	p53-Binding Protein 1
ATM	Ataxia Telangiectasia Mutated
ATR	Ataxia Telangiectasia and Rad3-Related
AUC	Area Under the Curve
BER	Base Excision Repair
BLISS	Breaks Labeling In Situ and Sequencing
BRCA1/2	Breast Cancer Gene 1/2
CLIA	Clinical Laboratory Improvement Amendments
CRM	Certified Reference Material
CtIP	C-terminal Binding Protein Interacting Protein
CV	Coefficient of Variation
DDR	DNA Damage Response
DMARD	Disease-Modifying Antirheumatic Drug
DMSO	Dimethyl Sulfoxide
DNA-PKcs	DNA-dependent Protein Kinase Catalytic Subunit
DRC	DNA Repair Capacity
DSB	Double-Strand Break
Fpg	Formamidopyrimidine DNA Glycosylase
GR	Glucocorticoid Receptor
H_2_O_2_	Hydrogen Peroxide
HCS	High-Content Screening
HCR	Host Cell Reactivation
hOGG1	Human 8-Oxoguanine DNA Glycosylase 1
HR	Homologous Recombination
HRD	Homologous Recombination Deficiency
IF	Immunofluorescence
IQC	Internal Quality Control
ISO	International Organization for Standardization
JRA	Juvenile Rheumatoid Arthritis
MDM2	Mouse Double Minute 2 Homolog
MFI	Mean Fluorescence Intensity
MIDRP	Minimum Information for DNA Repair Profiling
MRN	Mre11-Rad50-Nbs1 Complex
MS	Multiple Sclerosis
MTX	Methotrexate
MUTYH	MutY DNA Glycosylase
NEIL3	Nei-Like DNA Glycosylase 3
NER	Nucleotide Excision Repair
NGS	Next-Generation Sequencing
NHEJ	Non-Homologous End Joining
NSAID	Non-Steroidal Anti-Inflammatory Drug
Nth	Endonuclease III
PARP	Poly(ADP-Ribose) Polymerase
PBMC	Peripheral Blood Mononuclear Cell
PHA	Phytohemagglutinin
PI	Propidium Iodide
QC	Quality Control
RA	Rheumatoid Arthritis
RAD51	RAD51 Recombinase
RIF1	Replication Timing Regulatory Factor 1
ROS	Reactive Oxygen Species
RPA	Replication Protein A
SCGE	Single Cell Gel Electrophoresis
SLE	Systemic Lupus Erythematosus
SSB	Single-Strand Break
TBH	tert-Butyl Hydroperoxide
TNF-α	Tumor Necrosis Factor Alpha
UNG	Uracil DNA Glycosylase
WGS	Whole-Genome Sequencing
γH2AX	Phosphorylated Histone H2AX (Ser139)
